# The complete mitochondrial genome of the edible Basidiomycete mushroom *Thelephora ganbajun*

**DOI:** 10.1080/23802359.2017.1289344

**Published:** 2017-02-16

**Authors:** Pengfei Wang, Ying Zhang, Tao Sha, Jianping Xu

**Affiliations:** a State Key Laboratory for Conservation and Utilization of Bio-Resources in Yunnan, and Key Laboratory for Microbial Resources of the Ministry of Education, Yunnan University, Kunming, Yunnan, Peoples’ Republic of China;; b Department of Key Laboratory, The 2nd Affiliated Hospital of Kunming Medical University, Kunming, Yunnan, Peoples’ Republic of China;; c Department of Biology, McMaster University, Hamilton, Ontario, Canada

**Keywords:** *Thelephora ganbajun*, Thelephorales, polyporales, russulales, mitochondrial introns

## Abstract

The complete mitochondrial genome of the edible fungus *Thelephora ganbajun* was determined using Illumina sequencing. This mitogenome is a circular molecule of 52,857 bp in length with a GC content of 25.73%. Gene prediction showed that the mitogenome codes 28 tRNAs, 2 pseudo-tRNAs, and 21 known and 7 hypothetical proteins. The evolutionary relationships between *Th. ganbajun* and other representative species based on the mitogenome are consistent with those based on nuclear genes. The mitogenome information of *Th. ganbajun* should contribute to our understanding of the diversity and evolution of Thelephorales.

Thelephora species are basidiomycetes and ectomycorrhizal (ECM) fungi (Tedersoo et al. 2014). Members of this genus are distributed globally, contributing significantly to plant health and ecosystem stability (Kõljalg 1995; Martini & Hentic 2005; Yorou & Agerer 2008). In Yunnan province of China, *Thelephora ganbajun* is among the best-known *Thelephora* species, distributes primarily in pine forests and has been heavily harvested as a food delicacy (Zang 1986, 1987; Zhang & Yang 2013). While the nuclear genetic diversity and bioactive compounds of Th. ganbajun have been investigated (Lin & Ji-Kai 2001; Sha et al. 2008; Yang et al. 2004), little is known about its mitochondrial genome. Here we report the complete mitogenome sequence of Th. ganbajun (KY245891) and provide a phylogenetic analysis of its relationships with several representative taxa based on concatenated mitochondrial protein-coding genes.

The mitogenome was extracted from the whole genome sequence of a pure culture of strain P2 (collected in Yunnan province) using the Illumina HiSeq-1TB platform. This strain has been deposited in State Key Laboratory for Conservation and Utilization of Bio-Resources at Yunnan University. To obtain the mitogenome, each fastq file was QC filtered and subsequently assembled using Velvet (Zerbino & Birney 2008). The resulting assembly was used to create a long mate-pair library with insert 3000 ± 300 bp which was further assembled with the original Illumina library using AllPathsLG (Gnerre et al. 2011), to produce a 176.8 × coverage main assembly containing 3 scaffolds. The gaps were filled by separate PCR and sequencing with primers on regions flanking the gaps, resulting in one circular mitochondrial genome. Annotation was performed using MFannot (http://megasun.bch.umontreal.ca/cgi-bin/mfannot/mfannotInterface.pl) and the tRNAs and rRNAs were confirmed using RNAweasel (www.megasun.bch.umontreal.ca/RNAweasel/) and tRNAscan-SE (http://lowelab.ucsc.edu/tRNAscan-SE/) (Schattner et al. 2005). The general representation of the circular mitochondrial genome and the GC skew were prepared using the DNAPlotter software (Carver et al. 2009). The completely annotated mitogenome sequence is available in GenBank (accession KY245891).

The assembled mitochondrial genome was 52,857 bp in length with a GC content of 25.73%, coding for 28 tRNAs, 2 pseudo-tRNAs, 21 known proteins (including one pseudo gene and two overlapped polB2 genes), and 7 hypothetical proteins (or ORFs). The 28 tRNA genes covered 18 standard amino acids but without tRNA genes for Cysteine and Glutamic acid. Introns were found in three genes: nrDNA-LSU (2 group I A introns and 2 group I B introns), NAD5 (1 group I B intron) and COX1 (1 group I A intron and 1 group I B intron). Most introns have an ORF either of unknown function or code for a homing endonuclease.

Our mitogenome is the first submitted Thelephorales mitogenome in the GenBank database. Based on the concatenated protein sequences, our analyses revealed that *Th. ganbajun* was a member of Agaricomycotina and closely related to Polyporales and Russulales (Figure 1), consistent with the results obtained based on nuclear genes (Binder & Hibbett 2002; Garcia-Sandoval et al. 2011; Hibbett et al. 2007).

**Figure 1.**
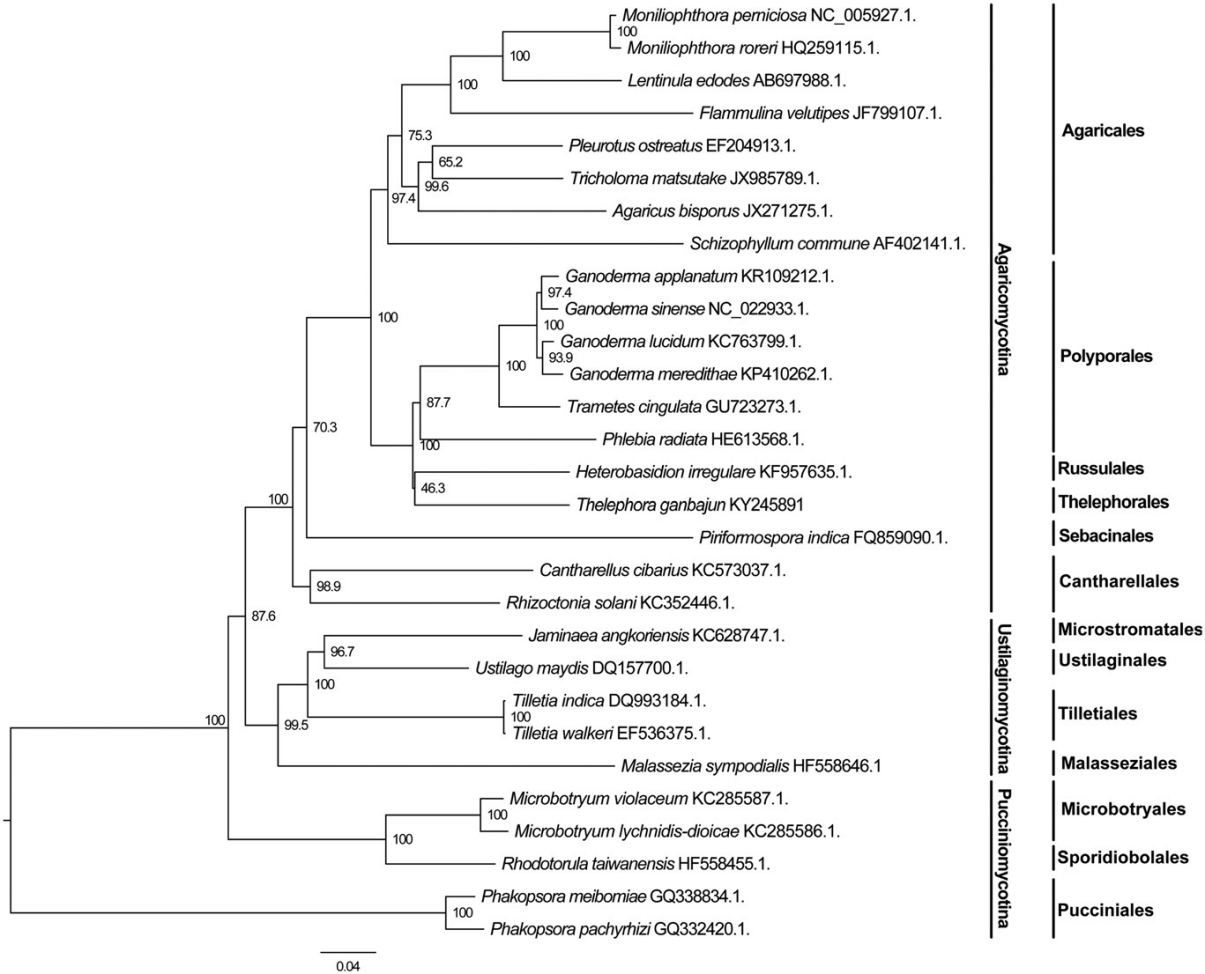
Phylogenetic analysis of 19 species of Agaricomycotina constructed using the Neighbour-Joining method as implemented in MEGA7.0 (Kumar et al. 2016) based on concatenated amino acid sequences of 14 mitochondrial protein-coding genes. The following 14 mitochondrial protein-coding genes were concatenated: atp6, atp8, atp9, cytb, cox1, cox2, cox3, nad1, nad2, nad3, nad4, nad4L, nad5 and nad6. The concatenated amino acid sequences were aligned using Clustal X (Thompson et al. 1997). The percentages of replicate trees in which the associated taxa clustered together in the bootstrap test (1000 replicates) were shown next to the branches.

## References

[CIT0001] Binder M, Hibbett DS. 2002 Higher-level phylogenetic relationships of homobasidiomycetes (mushroom-forming fungi) inferred from four rDNA regions. Mol Phylogenet Evol. 22:76–90. 11796031 10.1006/mpev.2001.1043

[CIT0002] Carver T, Thomson N, Bleasby A, Berriman M, Parkhill J. 2009 DNAPlotter: circular and linear interactive genome visualization. Bioinformatics. 25:119–120. 18990721 10.1093/bioinformatics/btn578 PMC2612626

[CIT0003] Garcia-Sandoval R, Wang Z, Binder M, Hibbett DS. 2011 Molecular phylogenetics of the Gloeophyllales and relative ages of clades of Agaricomycotina producing a brown rot. Mycologia. 103:510–524. 21186327 10.3852/10-209

[CIT0004] Gnerre S, MacCallum I, Przybylski D, Ribeiro FJ, Burton JN, Walker BJ, Sharpe T, Hall G, Shea TP, Sykes S. 2011 High-quality draft assemblies of mammalian genomes from massively parallel sequence data. Proc Natl Acad Sci USA. 108:1513–1518. 21187386 10.1073/pnas.1017351108 PMC3029755

[CIT0005] Hibbett DS, Binder M, Bischoff JF, Blackwell M, Cannon PF, Eriksson OE, Huhndorf S, James T, Kirk PM, Lücking R. 2007 A higher-level phylogenetic classification of the Fungi. Mycol Res. 111:509–547. 17572334 10.1016/j.mycres.2007.03.004

[CIT0006] Kõljalg U. 1995 *Tomentella* (Basidiomycota) and related genera in temperate Eurasia. Synopsis Fungorum, Fungiflora, Oslo 9:1–213.

[CIT0007] Kumar S, Stecher G, Tamura K. 2016 MEGA7: molecular evolutionary genetics analysis version 7.0 for bigger datasets. Mol Biol Evol. 33:1870–1874. 27004904 10.1093/molbev/msw054 PMC8210823

[CIT0008] Lin H, Ji-Kai L. 2001 Two novel phenylacetoxylated p-terphenyls from *Thelephora ganbajun* Zang. Z Naturforsch C J Biosci. 56:983–987. 11837688 10.1515/znc-2001-11-1213

[CIT0009] Martini E, Hentic R. 2005 *Tomentella lilacinogrisea* and *T. guadelupensis* sp. nov. Bull Soc Mycol Fr. 121:17–27.

[CIT0010] Schattner P, Brooks AN, Lowe TM. 2005 The tRNAscan-SE, snoscan and snoGPS web servers for the detection of tRNAs and snoRNAs. Nucleic Acids Res. 33:W686–W689. 15980563 10.1093/nar/gki366 PMC1160127

[CIT0011] Sha T, Xu J, Palanichamy MG, Zhang HB, Li T, Zhao ZW, Zhang YP. 2008 Genetic diversity of the endemic gourmet mushroom *Thelephora ganbajun* from south-western China. Microbiology. 154:3460–3468. 18957599 10.1099/mic.0.2008/020495-0

[CIT0012] Tedersoo L, Harend H, Buegger F, Pritsch K, Saar I, Kõljalg U. 2014 Stable isotope analysis, field observations and synthesis experiments suggest that Odontia is a non-mycorrhizal sister genus of Tomentella and Thelephora. Fungal Ecol. 11:80–90.

[CIT0013] Thompson JD, Gibson TJ, Plewniak F, Jeanmougin F, Higgins DG. 1997 The CLUSTAL_X windows interface: flexible strategies for multiple sequence alignment aided by quality analysis tools. Nucleic Acids Res. 25:4876–4882. 9396791 10.1093/nar/25.24.4876 PMC147148

[CIT0014] Yang WM, Liu JK, Hu L, Dong ZJ, Wu WL, Chen ZH. 2004 Antioxidant properties of natural p-terphenyl derivatives from the mushroom *Thelephora ganbajun*. Z Naturforsch C J Biosci. 59:359–362. 18998401 10.1515/znc-2004-5-612

[CIT0015] Yorou NS, Agerer R. 2008 Tomentella africana, a new species from Benin (West Africa) identified by morphological and molecular data. Mycologia. 100:68–80. 18488353 10.3852/mycologia.100.1.68

[CIT0016] Zang M. 1986 Criticism on *Thelephora ganbajun* position. Edible Fungi. 4:1–2.

[CIT0017] Zang M. 1987 Some new and noteworthy higher fungi from eastern Himalayas. Acta Bot Yunnanica. 1:009.

[CIT0018] Zerbino DR, Birney E. 2008 Velvet: algorithms for de novo short read assembly using de Bruijn graphs. Genome Res. 18:821–829. 18349386 10.1101/gr.074492.107 PMC2336801

[CIT0019] Zhang Q, Yang X. 2013 Situation, challenge and strategy of wild edible mushrooms export in Yunnan province. Technol Market. 20:316–318.

